# Supplementary material for: Retrospective cohort study to evaluate medication use in patients hospitalised with COVID-19 in Scotland: protocol for a national observational study
Source: BMJ Open. 2021 Nov 19;11(11):e054861. doi: 10.1136/bmjopen-2021-054861 (PMC8609490; doi:10.1136/bmjopen-2021-054861)
Supplement: Author's manuscript [file bmjopen-2021-054861.draft_revisions.pdf]

BMJ Open

BMJ Open is committed to open peer review. As part of this commitment we make the peer review history of every article we publish publicly available.

When an article is published we post the peer reviewers' comments and the authors' responses online. We also post the versions of the paper that were used during peer review. These are the versions that the peer review comments apply to.

The versions of the paper that follow are the versions that were submitted during the peer review process. They are not the versions of record or the final published versions. They should not be cited or distributed as the published version of this manuscript.

BMJ Open is an open access journal and the full, final, typeset and author-corrected version of record of the manuscript is available on our site with no access controls, subscription charges or pay-per-view fees (http://bmjopen.bmj.com).

If you have any questions on BMJ Open's open peer review process please email info.bmjopen@bmj.com

BMJ Open

Retrospective cohort study to evaluate medication use in patients hospitalised with COVID-19 in Scotland: protocol for a national observational study

Journal:	*BMJ Open*
Manuscript ID	bmjopen-2021-054861
Article Type:	Protocol
Date Submitted by the Author:	24-Jun-2021
Complete List of Authors:	Mueller, Tanja; University of Strathclyde, Strathclyde Institute of Pharmacy and Biomedical Sciences; Public Health Scotland Glasgow Office<br>Kerr, Steven; The University of Edinburgh, Usher Institute<br>McTaggart, Stuart; Public Health Scotland<br>Kurdi, Amanj; University of Strathclyde, Strathclyde Institute of Pharmacy and Biomedical Sciences; Public Health Scotland Glasgow Office<br>Vasileiou, Eleftheria; The University of Edinburgh, Usher Institute<br>Docherty, Annemarie; The University of Edinburgh, Usher Institute<br>Fraser, Kenny; Triscribe Limited<br>Shi, Ting; The University of Edinburgh, Usher Institute<br>Simpson, Colin; Victoria University of Wellington, School of Health, Wellington Faculty of Health; The University of Edinburgh, Usher Institute<br>Bennie, Marion; University of Strathclyde, Institute of Pharmacy and Biomedical Sciences; Public Health Scotland<br>Sheikh, Aziz; The University of Edinburgh, Usher Institute; HDR UK, BREATHE Hub
Keywords:	COVID-19, THERAPEUTICS, CLINICAL PHARMACOLOGY

SCHOLARONE™
Manuscripts

I, the Submitting Author has the right to grant and does grant on behalf of all authors of the Work (as defined in the below author licence), an exclusive licence and/or a non-exclusive licence for contributions from authors who are: i) UK Crown employees; ii) where BMJ has agreed a CC-BY licence shall apply, and/or iii) in accordance with the terms applicable for US Federal Government officers or employees acting as part of their official duties; on a worldwide, perpetual, irrevocable, royalty-free basis to BMJ Publishing Group Ltd ("BMJ") its licensees and where the relevant Journal is co-owned by BMJ to the co-owners of the Journal, to publish the Work in this journal and any other BMJ products and to exploit all rights, as set out in our licence.

The Submitting Author accepts and understands that any supply made under these terms is made by BMJ to the Submitting Author unless you are acting as an employee on behalf of your employer or a postgraduate student of an affiliated institution which is paying any applicable article publishing charge ("APC") for Open Access articles. Where the Submitting Author wishes to make the Work available on an Open Access basis (and intends to pay the relevant APC), the terms of reuse of such Open Access shall be governed by a Creative Commons licence – details of these licences and which Creative Commons licence will apply to this Work are set out in our licence referred to above.

Other than as permitted in any relevant BMJ Author's Self Archiving Policies, I confirm this Work has not been accepted for publication elsewhere, is not being considered for publication elsewhere and does not duplicate material already published. I confirm all authors consent to publication of this Work and authorise the granting of this licence.

For peer review only

Retrospective cohort study to evaluate medication use in patients hospitalised with COVID-19 in Scotland: protocol for a national observational study

Authors

Tanja Mueller[1,2], Steven Kerr[3], Stuart McTaggart[4], Amanj Kurdi[1,2], Eleftheria Vasileiou[3], Annemarie Docherty[3], Kenny Fraser[5], Ting Shi[3], Colin R Simpson[3,6], Marion Bennie[1,4], Aziz Sheikh[3,7]

Affiliations

[1]Strathclyde Institute of Pharmacy and Biomedical Sciences, University of Strathclyde, Glasgow, UK

[2]Public Health Scotland, Glasgow, UK

[3]Usher Institute, University of Edinburgh, Edinburgh, UK

[4]Public Health Scotland, Edinburgh, UK

[5]Triscribe Ltd, Glasgow, UK

[6]School of Health, Wellington Faculty of Health, Victoria University of Wellington, Wellington, NZ

[7]HDR UK BREATHE Hub, Edinburgh, UK

Corresponding author

Tanja Mueller, Strathclyde Institute of Pharmacy and Biomedical Sciences, University of Strathclyde, 161 Cathedral Street, Glasgow G4 0RE, UK; email tanja.muller@strath.ac.uk

ORCID

Tanja Mueller: 0000-0002-0418-4789

Amanj Kurdi: 0000-0001-5036-1988

Eleftheria Vasileiou: 0000-0001-6850-7578

Annemarie Docherty: 0000-0001-8277-420X

Ting Shi: 0000-0002-4101-4535

Colin Simpson: 0000-0002-5194-8083

Marion Bennie: 0000-0002-4046-629x

Aziz Sheikh: 0000-0001-7022-3056

Key words: COVID-19, hospital treatment, drug utilisation, dexamethasone, remdesivir, tocilizumab

Word count: abstract 291; main text 2376 (excluding abstract, tables and references)

For peer review only

Abstract

Introduction: Coronavirus disease 2019 (COVID-19) has caused millions of hospitalisations and deaths globally. A range of vaccines have been developed and are being deployed at scale in the UK to prevent SARS-CoV-2 infection, which have reduced risk of infection and severe COVID-19 outcomes. Those with COVID-19 are now being treated with several repurposed drugs based on evidence emerging from recent clinical trials. However, there is currently limited real-world data available related to the use of these drugs in routine clinical practice. The purpose of this study is to address the prevailing knowledge gaps regarding the use of dexamethasone, remdesivir, and tocilizumab by conducting an exploratory drug utilisation study, aimed at providing in-depth descriptions of patients receiving these drugs as well as the treatment patterns observed in Scotland.

Methods and analysis: Retrospective cohort study, comprising adult patients admitted to hospital with confirmed or suspected COVID-19 across five Scottish Health Boards using data from in-hospital ePrescribing linked to the Early Estimation of Vaccine and Anti-Viral Effectiveness (EAVE II) COVID-19 surveillance platform. The primary outcome will be exposure to the medicines of interest (dexamethasone, remdesivir, tocilizumab), either alone or in combination; exposure will be described in terms of drug(s) of choice; prescribed and administered dose; treatment duration; and any changes in treatment, e.g. dose escalation and/or switching to an alternative drug. Analyses will primarily be descriptive in nature.

Ethics and dissemination: Ethical and information governance approvals have been obtained by the National Research Ethics Service Committee, South East Scotland 02, and the Public Benefit and Privacy Panel for Health and Social Care, respectively. Findings from this study will be presented at academic and clinical conferences, and to the funders and other interested parties as appropriate; study findings will also be published in peer-reviewed journals.

For peer review only - http://bmjopen.bmj.com/site/about/guidelines.xhtml

Strengths and limitations of this study

- This study will use data collected as part of routine care to address prevailing knowledge gaps with regards to the treatment of hospitalised COVID-19 patients.
- In-patient electronic prescribing data will be linked with a wide range of other datasets, enabling an in-depth description of current clinical practice in Scotland.
- Analyses will be descriptive in nature; causal analyses will be outwith the scope of this study due to its observational nature.

For peer review only

For peer review only - http://bmjopen.bmj.com/site/about/guidelines.xhtml

INTRODUCTION

Since first appearing in Wuhan, China, in late 2019, the new “severe acute respiratory syndrome coronavirus 2” (SARS-CoV-2) has spread globally, resulting in the World Health Organization (WHO) first declaring a Public Health Emergency of International Concern and then, in March 2020, a pandemic.[1] The disease caused by SARS-CoV-2 is now widely known as “coronavirus disease 2019”, or COVID-19.

Early symptoms of COVID-19 tend to occur between 5 – 10 days after infection, and commonly include fever, loss of smell and/or taste, and a persistent cough.[2] Symptoms may become increasingly severe over a period of approximately two weeks, and can lead to hospitalisation mainly due to breathing problems; patients with severe disease frequently require mechanical ventilation.[3] COVID-19 potentially leads to organ damage, and can result in long-term health problems (“long COVID”).[4] Disease outcomes generally appear to be linked to age and pre-existing conditions, including cardiovascular diseases and diabetes.[5]

A number of COVID-19 vaccines have been developed and are now being successfully deployed at scale in the UK.[6,7] Furthermore, while the condition itself is self-limiting in the majority of cases, a range of repurposed drugs are currently being used to alleviate symptoms and/or decrease mortality in hospitalised COVID-19 patients, mostly based on evidence emerging from clinical trials – including, e.g., antiviral drugs that have previously been tested in conditions caused by similar viruses such as SARS or MERS (Middle East respiratory syndrome) or other viral infections such as HIV or Ebola;[8,9] anti-inflammatory drugs including corticosteroids[10] and monoclonal antibodies;[11–14] and a raft of other drugs, from antibiotics[15] to interferons.[16] In addition, convalescent plasma therapy has been proposed.[17]

With interest in this area remaining high, new study results being reported on a frequent basis, and several clinical trials still ongoing, treatment recommendations are rapidly updated;[18] therefore, treatment guidelines – and, consequently, clinical practice – are likely to differ substantially, both across countries and over time. For instance, a recent multi-national cohort study has investigated the use of repurposed and adjuvant drugs in hospitalised COVID-19 patients in China, South Korea, Spain, and the United States, and found that azithromycin, the antivirals lopinavir and ritonavir, and the anti-malaria drug hydroxychloroquine were frequently used at the beginning of the pandemic; however, following reports of the non-effectiveness of these drugs in combination with safety issues related to

For peer review only - http://bmjopen.bmj.com/site/about/guidelines.xhtml

hydroxychloroquine, their use has declined, and dexamethasone and remdesivir use have instead been increasing. In addition, use patterns differed considerably between these countries.[19]

Dexamethasone,[20] remdesivir,[21] and tocilizumab[22] have been recommended for use in hospitalised patients with severe COVID-19 within the UK based on randomised controlled trial evidence, most prominently the RECOVERY trial.[23] There is, however, currently limited real-world evidence available related to the use of these drugs in routine clinical practice. For instance, it is thus far unclear which patients are being prescribed dexamethasone, remdesivir, and/or tocilizumab as part of their in-hospital treatment, and at what point; what the most common treatment patterns are; how the use of these drugs has changed since the start of the pandemic; and whether there are any geographical differences observable. Further evidence on the real-world clinical effectiveness and safety of these drugs is also required.[24]

There have been few published studies of in-hospital drug utilisation. This has been due, in part, to patient-level data being unavailable as drug prescribing and administration records are paper-based in many secondary care settings.[25] The implementation of electronic prescribing in hospitals in Scotland has simplified data sharing across health care settings. The wider roll-out of the "Hospital Electronic Prescribing and Medicines Administration" (HEPMA) system was initiated in 2014[26] in line with the Scottish eHealth strategy,[27] and HEPMA is now available to hospitals across five out of the 14 Health Boards in Scotland (regional organisations responsible for delivering health care to their respective populations).[28]

Our aim is to contribute to addressing the prevailing knowledge gaps by conducting an exploratory drug utilisation study using data from in-hospital ePrescribing linked to the Early Estimation of Vaccine and Anti-Viral Effectiveness (EAVE II) COVID-19 surveillance platform.[24] This linked data will be analysed to provide an in-depth description of the treatments hospitalised COVID-19 patients receive (whether alone or in combination), and to describe the outcomes in these patients. The drugs of interest include dexamethasone, remdesivir, and tocilizumab, based on information requests by clinicians working in this setting; these drugs are currently routinely used in patients hospitalised with COVID-19 in Scotland due to recent treatment recommendations.

For peer review only - http://bmjopen.bmj.com/site/about/guidelines.xhtml

Primary objectives

- Identify patients being treated with dexamethasone, remdesivir, and/or tocilizumab (either as monotherapy or in combination) for COVID-19 after being admitted to hospital as part of standard care;
- Describe and summarise baseline characteristics of these patients, including COVID-19 status (suspected at hospital admission based on symptoms, vs confirmed via polymerase chain reaction (PCR) test); socio-demographics (age; sex; Health Board; deprivation; hospital type; admission from care home; hospital readmission); and clinical variables potentially related to treatment choice and/or possible outcomes (including, but not restricted to, comorbidities,[29] concomitant medication, and Intensive Care Unit (ICU) admission);
- Describe treatment patterns, including drug chosen and dose administered; treatment duration; dose escalations; and changes in the drug given (e.g. switching from dexamethasone to hydrocortisone or methylprednisolone);
- Describe patterns of medicines use over time and across geographical areas, and potentially by patient characteristic as and when appropriate;
- Map out patient pathways and describe admission episodes and their outcomes (hospital admission details and duration of in-hospital stay, ICU transferal, administration of dexamethasone or any other drug of interest, discharge or death); potentially stratified by patient characteristics as and when appropriate.

Secondary objective

- Evaluate the impact of guideline changes on the patterns of use of dexamethasone, remdesivir, and tocilizumab over time.

METHODS AND ANALYSIS

Study design

Retrospective cohort study, comprising adult patients (18 years of age or older) admitted to hospital with confirmed or suspected COVID-19 across five Scottish Health Boards: NHS Ayrshire & Arran, NHS

For peer review only - http://bmjopen.bmj.com/site/about/guidelines.xhtml

Dumfries & Galloway, NHS Forth Valley, NHS Lanarkshire, and NHS Lothian. The total population size of these Health Boards was approximately 2.4m people (~45% of the Scottish population) in mid-2019.[30] However, since implementation of HEPMA in NHS Lothian happened later than in the other Health Boards, data might not be as complete, particularly for the early months of the study period.

Data sources

The data to be used for this study is part of the EAVE II platform, which has been implemented to determine COVID-19 related risk factors and the COVID-19 health care burden; and to evaluate the uptake, safety, and effectiveness of therapeutic interventions.[24] All data have been collected as part of routine care.

The EAVE II data source contains primary health care records, linked with patient-level secondary care data using the Community Health Index (CHI) number[31] – a unique patient identifier used throughout the Scottish health system – and comprises the following datasets:

- COVID-19 test results: Electronic Communication of Surveillance Scotland (ECOSS)[32]
- Vaccination status: Turas Vaccine Management Tool (TVMT),[33] GP extract
- Hospital admissions and in-patient episodes: Scottish Morbidity Record (SMR01), Rapid Preliminary Inpatient Data (RAPID), and Scottish Intensive Care Society Audit Group database (SICSAG)[34]
- In-hospital medicines use and community prescriptions: HEPMA[35] and Prescribing Information System (PIS)[34], respectively
- Mortality: National Records of Scotland (NRS)[34]

HEPMA will be used for identification of the main study outcomes, including medication use patterns; all available datasets may be used to identify other study outcomes as appropriate and feasible.

EAVE II data are held by Public Health Scotland; pseudonymised data will be accessed using the National Safe Haven, a secure, closed environment.[36]

For peer review only - http://bmjopen.bmj.com/site/about/guidelines.xhtml

Study population

The study population will comprise all adult patients admitted to hospital in the five aforementioned Health Boards since 01.03.2020 with a primary diagnosis of COVID-19, up to the latest date available. COVID-19 hospitalisations will be defined as hospitalisations within 28 days of a positive PCR test, or based on an admission with an ICD-10 code for COVID-19 (U07.1 and U07.2) as recorded in hospital episode records (SMR01 and/or RAPID); ICD-10 diagnoses will be confirmed using available PCR test results (ECOSS) where possible. Hence, the population will include both laboratory confirmed and clinical based diagnosis, respectively.

Patients will be followed up from the index date, defined as the first prescribing date for any of the medications of interest (the exposure), until discharge from hospital, death, or the end of the study period subject to data availability, whichever occurs first.

Patients receiving any of the drugs as part of a clinical trial will be excluded for analyses based on trial participation information if available (e.g. trial flag in HEPMA); or based on the dates where drugs became recommended for use in daily practice as communicated by the Scottish Government/NHS Scotland (see also Table 1 below for details).

Primary outcome

The primary outcome will be treatment with the medicines of interest, either alone or in combination, with a particular emphasis on dexamethasone as dexamethasone is the most widely used of these drugs, and the availability of both prescribing and administration data is expected to be high. All patients receiving dexamethasone will be included in the first instance; however, analyses will mainly focus on those receiving the recommended dosing regimen for patients with COVID-10 (6mg po or 1.8ml iv, once daily for 10 days)[20] since other dosing regimens are more likely being prescribed for indications other than COVID-19. Alternative recommended corticosteroids such as prednisolone and hydrocortisone will also be considered (recommended doses: 40mg po once daily for 10 days; 50mg iv every eight hours for 10 days, respectively). Remdesivir and tocilizumab will be included for analyses where sufficient data are available; since these two drugs are given intravenously, the data available might be limited (i.e. exclude the exact dose administered).

Exposure will be described in terms of drug(s) of choice; prescribed and administered dose; treatment duration; and any changes in treatment, e.g. dose escalation and/or switching to an alternative drug

For peer review only - http://bmjopen.bmj.com/site/about/guidelines.xhtml

(including time to dose escalation/switching and reasons for these, if available). The first drug prescribed on HEPMA following admission to hospital on or after the date of (possible) COVID-19 diagnosis will be defined as the index drug (i.e. dexamethasone, remdesivir, or tocilizumab); the date of the first recorded prescription will be used as the index date (for the purpose of setting the baseline). Duration of treatment will be calculated using the dates of first/last recorded administration of the drug in question.

Other outcomes

Additional outcomes relating to the primary study objective include hospital specialty at admission, in-hospital transfer (e.g. admission to ICU), length of stay, and outcome of hospital episode (discharge or death).

Secondary outcomes include in-hospital mortality, i.e. death on the same day as discharge; and out-of-hospital mortality following discharge, if feasible.

Covariates

Patient characteristics of interest that might potentially influence choice of drug, duration of treatment, and (possibly) treatment outcomes will be identified and summarised at baseline, and comprise socio-demographic factors (age, sex, Health Board of residence, level of deprivation); disease-related aspects; comorbidities; and concomitant medication.

The level of deprivation will be characterised using the Scottish Index of Multiple Deprivation (SIMD), an area index combining information with regards to health, access to services, education, employment, income, housing, and crime.[37] Disease-related aspects refer to information potentially linked to disease severity, e.g. level of hospital care/additional treatments received (ICU admission, mechanical ventilation) if and where available; while O_2 saturation levels would be highly relevant, particularly with regards to treatment outcomes, this information is not present in the available dataset. Comorbidities of interest will comprise mainly those conditions used to identify patients at high risk of adverse outcomes (i.e. shielding list),[29] e.g. respiratory disease (asthma, chronic obstructive pulmonary disease (COPD)), cardiovascular diseases, diabetes (type 1 and type 2), chronic kidney disease, and cancer; other comorbidities might also be included. Concomitant medication at baseline will focus on drugs potentially impacting the immune system and/or affecting the risk of infections (immuno-suppressants, steroids, antimicrobial drugs), and those with an (hypothesised) effect on disease severity or outcome – either directly or as a proxy for underlying conditions potentially not captured otherwise within the dataset

For peer review only - http://bmjopen.bmj.com/site/about/guidelines.xhtml

(e.g. long-acting muscarinic antagonists (LAMA)/long acting beta-agonists (LABA), insulin and anti-diabetic drugs, anticoagulants, antiplatelet drugs, angiotensin-converting enzyme (ACE) inhibitors, angiotensin receptor blockers (ARBs)). If possible, COVID-19 vaccination status of patients will also be assessed. Furthermore, the presence of polypharmacy – defined as the simultaneous use of five or more different medications prior to being admitted to hospital – will be identified.

Baseline characteristics will be defined using all available data, with restrictions on included time periods (mainly with regards to concomitant medication) based on the specifications used in previous studies.[5,6,24]

Exposure, outcome, and relevant covariates – alongside the data source, their coding, and a brief description – are presented in table 1.

Table 1: Description of variables (cohort identification, outcomes, covariates)

**Variable**	**Data source**	**Description**	**Value**
Cohort: COVID-19 status			
Cause of admission	SMR01/ RAPID	Suspected or confirmed	ICD10 codes: U07.1, U07.2
PCR test result	ECOSS	COVID-19 test result (within 28 days prior to admission)	Categorical: Positive, negative, unavailable
Primary outcome: medication use			
Drug name	HEPMA	Drugs of interest: Dexamethasone, remdesivir, tocilizumab [1]	Character (name, according to dm+d)
Drug dose	HEPMA	Prescribed and administered	Numeric (mg, ml)
Prescribed date	HEPMA	First prescribed date: index date	Date (yyyy-mm-dd)
Administered date	HEPMA	Dates of drug administration	Date (yyyy-mm-dd)
Duration of treatment *	HEPMA	(first – last administered date) [2]	Numeric (days)
Treatment changes *	HEPMA	Changes in dosing and/or drug	Categorical: yes, no
Secondary outcomes			
Hospital specialty	SMR01	At admission	Character (name)
Specialty changes *	SMR01	Internal transferals during stay	Categorical: yes, no

For peer review only - http://bmjopen.bmj.com/site/about/guidelines.xhtml

ICU/HDU	SICSAG	Admission to intensive care	Categorical: yes, no
Discharge: alive	SMR01	Outcome of hospital episode	Categorical: home, w/family, care facility, other hospital
Discharge: dead	SMR01, NRS	Outcome of hospital admission (In-hospital mortality)	Categorical: yes, no<br>Cause: ICD-10 codes
Death	NRS	Overall mortality (after discharge)	Categorical: yes, no<br>Cause: ICD-10 codes
Length of stay [3]	SMR01	Duration of in-hospital stay	Numeric (days)
Covariates: socio-demographic			
Age *	GP extract	Patient age at index date	Numeric (years)
Sex	GP extract	Biological sex at birth	Categorical: male, female
Health Board	GP extract	Patient place of residence at admission	Categorical: A&A, D&G, Forth Valley, Lanarkshire, Lothian;
Data zone	GP extract	Patient place of residence	Categorical
SIMD	GP extract	Level of deprivation, based on data zone of residence	Categorical: 1 (most) to 5 (least deprived)
Covariates: disease (severity) related			
COVID-19 vaccination status	GP extract/ TVMT	Status at hospital admission	Categorical: unvaccinated, vaccinated once, twice
Level of care	SICSAG/ SMR01	Admission to ICU; level of care received while at ICU	Categorical: yes, no<br>Categorical: ACP levels 0-3
Supporting medication	HEPMA	Therapeutics prescribed and administered during in-hospital stay	Character (name, according to dm+d)
Covariates: comorbidities and concomitant medication			
Other causes of admission	SMR01	Conditions underlying or attributing to hospital admission	ICD-10 codes
Comorbidities	GP extract/ SMR01	Pre-existing conditions	READ codes, ICD-10 codes
Charlson score * [38]	SMR01	Estimated based on secondary care data (historic hospital episodes)	Numeric
Concomitant medication	PIS	Potential proxy for comorbidities; specific drugs of interest	Character (name, according to the BNF)
Polypharmacy *	PIS	Based on number of different drugs prescribed simultaneously	Categorical: yes, no

For peer review only

For peer review only - http://bmjopen.bmj.com/site/about/guidelines.xhtml

* denotes derived variables

[1] Cut-off dates to exclude patients who have been treated as part of a clinical trial, if no trial flag participation available in the dataset: remdesivir 29.05.2020; dexamethasone16.06.2020; tocilizumab 08.01.2021
[2] Adding discharge/outpatient prescribing if patient discharged prior to end of treatment regimen (if available)
[3] Can be derived if variable not readily available in dataset (date of discharge – first date of admission)

ACP - Augmented Care Period; BNF - British National Formulary; dm+d - Dictionary of Medicines and Devices; ECOSS - Electronic Communication of Surveillance in Scotland; HDU - High Dependency Unit; HEPMA - Hospital Electronic Prescribing and Medicines Administration; ICD-10 - International Classification of Diseases, 10th Edition; ICU - Intensive Care Unit; NRS - National Records of Scotland; PCR - polymerase chain reaction; PIS - Prescribing Information System; RAPID - rapid preliminary in-patient data; SICSAG - Scottish Intensive Care Society Audit Group; SIMD - Scottish Index of Multiple Deprivation; SMR01 - Scottish Morbidity Records, inpatient dataset; TVMT - Tuas Vaccine Management Tool

Statistical analysis

All analyses relating to the primary objectives of this study will be descriptive in nature, and may include counts/frequencies for categorical variables, and mean/SD or median/IQR for continuous variables, as appropriate. In addition, patient pathways will be visualised using Sankey plots or similar techniques.

The impact of changes in treatment guidelines on the use of dexamethasone will be evaluated using interrupted time series analysis; logistic regression or time-to-event analysis (e.g. Kaplan-Meier plots) will be used to assess discharge patterns or patient mortality, if feasible.

All analyses will be conducted using R/RStudio, version 3.6.1.[39,40]

Patient and public involvement

The EAVE II Public Advisory group are a diverse group of PPI contributors who meet monthly to incorporate the views of patients and the public into research using the EAVE II dataset. This includes shaping of research via the EAVE II Steering Group, which is attended by our two lay leads. The lay summary for this research will be co-written with our PPI contributors and shared via the outputs section of the EAVE II website,[36] hosted by the University of Edinburgh.

ETHICS AND DISSEMINATION

Ethical and information governance approvals have been obtained by the National Research Ethics Service Committee (REC), South East Scotland 02 (REC number: 12/SS/0201), and the Public Benefit and Privacy Panel for Health and Social Care (reference number: 1920-0279) respectively. Findings from this study will be presented at academic and clinical conferences, and to the funders and other interested parties as appropriate. Study findings will also be published in peer-reviewed journals; reporting of

For peer review only - http://bmjopen.bmj.com/site/about/guidelines.xhtml

findings will follow the STROBE (Strengthening the Reporting of Observational Studies in Epidemiology)[41] and RECORD (Reporting of Studies conducted using Observational Routinely-collected Data) guidelines.

Acknowledgements: We thank Dave Kelly from Albasoft Ltd for his support with making primary care data available; and Wendy Inglis-Humphrey and Vicky Hammersley for their support with project management and administration. This work is only possible because of the wealth of information collected by the NHS as part of routine clinical practice.

Authors' contributions: TM, SK, SM, AK and MB conceptualised the study. EV, AD, KF, TS and CS provided additional methodological and/or clinical advice. AS is the principal investigator of the EAVE II project and provides strategic advice. TM drafted the protocol. All authors read, critically revised, and approved the final draft.

Funding: This analysis is part of the Early Assessment of COVID-19 epidemiology and Vaccine/anti-viral Effectiveness (EAVE II) study. EAVE II is funded by the Medical Research Council [MC_PC_19075] with the support of BREATHE – The Health Data Research Hub for Respiratory Health [MC_PC_19004], which is funded through the UK Research and Innovation Industrial Strategy Challenge Fund and delivered through Health Data Research UK. Additional support has been provided through the Scottish Government DG Health and Social Care.

Competing interests: AS reports grants from NIHR, grants from MRC, grants from HDR UK, during the conduct of the study. CS reports funding from NIHR (UK), MRC (UK), CSO (UK), Health Research Council (NZ), and Ministry for Business, Innovation and Employment (NZ) during the conduct of this study. KF is Director of Triscribe Ltd, a company providing data quality services and software support. All other authors report no conflicts of interest.

Data availability statement: NHS data is confidential, and is only available upon request subject to approval by a Caldicott Guardian/the Public Benefit and Privacy Panel for Health and Social Care.

Patient consent for publication: Not required.

For peer review only - http://bmjopen.bmj.com/site/about/guidelines.xhtml

REFERENCES

1 WHO. Coronavirus disease (COVID-19) – World Health Organization. 2021.https://www.who.int/emergencies/diseases/novel-coronavirus-2019 (accessed 8 Jun 2021).

2 NHS 24. Coronavirus (COVID-19) in Scotland | NHS inform. 2021.https://www.nhsinform.scot/illnesses-and-conditions/infections-and-poisoning/coronavirus-covid-19 (accessed 8 Jun 2021).

3 Kevadiya BD, Machhi J, Herskovitz J, *et al.* Pharmacotherapeutics of SARS-CoV-2 Infections. *J Neuroimmune Pharmacol* 2021;:1–26. doi:10.1007/s11481-020-09968-x

4 Iacobucci G. Long covid: Damage to multiple organs presents in young, low risk patients. *BMJ* 2020;**371**:m4470. doi:10.1136/bmj.m4470

5 Clift AK, Coupland CAC, Keogh RH, *et al.* Living risk prediction algorithm (QCOVID) for risk of hospital admission and mortality from coronavirus 19 in adults: national derivation and validation cohort study. *BMJ* 2020;**371**:m3731. doi:10.1136/bmj.m3731

6 Vasileiou E, Simpson CR, Shi T, *et al.* Interim findings from first-dose mass COVID-19 vaccination roll-out and COVID-19 hospital admissions in Scotland: a national prospective cohort study. *The Lancet* 2021;**397**:1646–57. doi:10.1016/S0140-6736(21)00677-2

7 Vasileiou E, Simpson CR, Robertson C, *et al.* Effectiveness of First Dose of COVID-19 Vaccines Against Hospital Admissions in Scotland: National Prospective Cohort Study of 5.4 Million People. Rochester, NY: : Social Science Research Network 2021. doi:10.2139/ssrn.3789264

8 Beigel JH, Tomashek KM, Dodd LE, *et al.* Remdesivir for the Treatment of Covid-19 — Final Report. *New England Journal of Medicine* Published Online First: 22 May 2020. doi:10.1056/NEJMoa2007764

9 Horby PW, Mafham M, Bell JL, *et al.* Lopinavir–ritonavir in patients admitted to hospital with COVID-19 (RECOVERY): a randomised, controlled, open-label, platform trial. *The Lancet* 2020;**396**:1345–52. doi:10.1016/S0140-6736(20)32013-4

10 RECOVERY Collaborative Group, Horby P, Lim WS, *et al.* Dexamethasone in Hospitalized Patients with Covid-19. *N Engl J Med* 2021;**384**:693–704. doi:10.1056/NEJMoa2021436

11 REMAP-CAP Investigators. Interleukin-6 Receptor Antagonists in Critically Ill Patients with Covid-19. *New England Journal of Medicine* 2021;**384**:1491–502. doi:10.1056/NEJMoa2100433

12 RECOVERY Collaborative Group, Horby PW, Pessoa-Amorim G, *et al.* Tocilizumab in patients admitted to hospital with COVID-19 (RECOVERY): preliminary results of a randomised, controlled, open-label, platform trial. *medRxiv* 2021;:2021.02.11.21249258. doi:10.1101/2021.02.11.21249258

13 Weinreich DM, Sivapalasingam S, Norton T, *et al.* REGN-COV2, a Neutralizing Antibody Cocktail, in Outpatients with Covid-19. *New England Journal of Medicine* 2021;**384**:238–51. doi:10.1056/NEJMoa2035002

14 RECOVERY Collaborative Group, Horby PW, Mafham M, *et al.* Casirivimab and imdevimab in patients admitted to hospital with COVID-19 (RECOVERY): a randomised, controlled, open-label, platform trial. *medRxiv* 2021;:2021.06.15.21258542. doi:10.1101/2021.06.15.21258542

For peer review only - http://bmjopen.bmj.com/site/about/guidelines.xhtml

15 Abaleke E, Abbas M, Abbasi S, *et al.* Azithromycin in patients admitted to hospital with COVID-19 (RECOVERY): a randomised, controlled, open-label, platform trial. *The Lancet* 2021;**397**:605–12. doi:10.1016/S0140-6736(21)00149-5

16 Monk PD, Marsden RJ, Tear VJ, *et al.* Safety and efficacy of inhaled nebulised interferon beta-1a (SNG001) for treatment of SARS-CoV-2 infection: a randomised, double-blind, placebo-controlled, phase 2 trial. *The Lancet Respiratory Medicine* 2021;**9**:196–206. doi:10.1016/S2213-2600(20)30511-7

17 Simonovich VA, Burgos Pratx LD, Scibona P, *et al.* A Randomized Trial of Convalescent Plasma in Covid-19 Severe Pneumonia. *New England Journal of Medicine* 2021;**384**:619–29. doi:10.1056/NEJMoa2031304

18 National Institute for Health and Care Excellence. Recommendations | COVID-19 rapid guideline: managing COVID-19 | Guidance | NICE. https://www.nice.org.uk/guidance/ng191/chapter/Recommendations (accessed 21 Jun 2021).

19 Prats-Uribe A, Sena AG, Lai LYH, *et al.* Use of repurposed and adjuvant drugs in hospital patients with covid-19: multinational network cohort study. *BMJ* 2021;**373**:n1038. doi:10.1136/bmj.n1038

20 Scottish Government. COVID-19 therapeutic alert: Dexamethasone in the treatment of COVID-19: implementation and management of supply for treatment in hospitals. https://www.sehd.scot.nhs.uk/publications/DC20200616COVID-19Dexamethasone.pdf (accessed 6 Aug 2021).

21 NHS England. Coronavirus » Interim Clinical Commissioning Policy: Remdesivir for patients hospitalised with COVID-19 (adults and children 12 years and older). https://www.england.nhs.uk/coronavirus/publication/interim-clinical-commissioning-policy-remdesivir-for-patients-hospitalised-with-covid-19-adults-and-children-12-years-and-older/ (accessed 23 Mar 2021).

22 NHS England. Coronavirus » Interim Clinical Commissioning Policy: Tocilizumab for hospitalised patients with COVID-19 pneumonia (adults). https://www.england.nhs.uk/coronavirus/publication/interim-clinical-commissioning-policy-tocilizumab-for-hospitalised-patients-patients-with-covid-19-pneumonia-adults/ (accessed 23 Mar 2021).

23 Nuffield Department of Population Health. RECOVERY - Randomised Evaluation of COVID-19 Therapy. 2021.https://www.recoverytrial.net/ (accessed 8 Jun 2021).

24 Simpson CR, Robertson C, Vasileiou E, *et al.* Early Pandemic Evaluation and Enhanced Surveillance of COVID-19 (EAVE II): protocol for an observational study using linked Scottish national data. *BMJ Open* 2020;**10**:e039097. doi:10.1136/bmjopen-2020-039097

25 Warren LR, Clarke J, Arora S, *et al.* Improving data sharing between acute hospitals in England: an overview of health record system distribution and retrospective observational analysis of inter-hospital transitions of care. *BMJ Open* 2019;**9**:e031637. doi:10.1136/bmjopen-2019-031637

26 NHS Scotland. HEPMA | eHealth. https://www.ehealth.scot/case-studies/hepma/ (accessed 8 Jun 2021).

27 Scottish Government. eHealth Strategy 2014-2017. Edinburgh, UK: : Scottish Government 2015. https://www.gov.scot/publications/ehealth-strategy-2014-2017/ (accessed 15 Oct 2020).

28 NHS Scotland. Organisations – Scotland’s Health on the Web. 2020.https://www.scot.nhs.uk/organisations/ (accessed 8 Jun 2021).

For peer review only

For peer review only - http://bmjopen.bmj.com/site/about/guidelines.xhtml

29 NHS Digital. Rule logic. NHS Digital. https://digital.nhs.uk/coronavirus/shielded-patient-list/methodology/rule-logic (accessed 7 Jun 2021).

30 Public Health Scotland. Population Estimates - Scottish Health and Social Care Open Data. 2021.https://www.opendata.nhs.scot/dataset/population-estimates (accessed 14 Jun 2021).

31 Information Services Division. Data Dictionary A-Z: CHI number. ISD Scotland Data Dictionary. https://www.ndc.scot.nhs.uk/Dictionary-A-Z/Definitions/index.asp?ID=128&Title=CHI%20Number (accessed 15 Oct 2020).

32 Health Protection Scotland. Data and surveillance. https://www.hps.scot.nhs.uk/data/ (accessed 8 Jun 2021).

33 NHS Education for Scotland. Turas Vaccination Management tool. 2021.https://learn.nes.nhs.scot/42708/turas-vaccination-management-tool (accessed 8 Jun 2021).

34 Information Services Division. National Data Catalogue: National Datasets. ISD Scotland National Data Catalogue. https://www.ndc.scot.nhs.uk/National-Datasets/index.asp (accessed 15 Oct 2020).

35 NHS Digital. Hospital Electronic Prescribing and Medicines Administration (HEPMA) Data - Scotland. NHS Digital. https://digital.nhs.uk/about-nhs-digital/corporate-information-and-documents/directions-and-data-provision-notices/data-provision-notices-dpns/electronic-prescribing-and-medicines-administration-data-scotland (accessed 8 Jun 2021).

36 University of Edinburgh. EAVE II. The University of Edinburgh. https://www.ed.ac.uk/usher/eave-ii (accessed 8 Jun 2021).

37 Scottish Government. The Scottish Index of Multiple Deprivation. Statistics. 2020.https://www2.gov.scot/SIMD (accessed 15 Oct 2020).

38 Quan H, Sundararajan V, Halfon P, *et al.* Coding algorithms for defining comorbidities in ICD-9-CM and ICD-10 administrative data. *Med Care* 2005;**43**:1130–9. doi:10.1097/01.mlr.0000182534.19832.83

39 R Core Team. *R: A language and environment for statistical computing*. Vienna, Austria: : R Foundation for Statistical Computing 2020. https://www.R-project.org/

40 RStudio Team. *RStudio: Integrated Development for R*. Boston, MA: : RStudio, PBC 2020. http://www.rstudio.com/

41 Elm E von, Altman DG, Egger M, *et al.* Strengthening the reporting of observational studies in epidemiology (STROBE) statement: guidelines for reporting observational studies. *BMJ* 2007;**335**:806–8. doi:10.1136/bmj.39335.541782.AD

For peer review only

For peer review only - http://bmjopen.bmj.com/site/about/guidelines.xhtml

Reporting checklist for cohort study.

Based on the STROBE cohort guidelines.

Instructions to authors

Complete this checklist by entering the page numbers from your manuscript where readers will find each of the items listed below.

Your article may not currently address all the items on the checklist. Please modify your text to include the missing information. If you are certain that an item does not apply, please write "n/a" and provide a short explanation.

Upload your completed checklist as an extra file when you submit to a journal.

In your methods section, say that you used the STROBE cohortreporting guidelines, and cite them as:

von Elm E, Altman DG, Egger M, Pocock SJ, Gotzsche PC, Vandenbroucke JP. The Strengthening the Reporting of Observational Studies in Epidemiology (STROBE) Statement: guidelines for reporting observational studies.

For peer review only

		Reporting Item	Page Number
**Title and abstract**			
Title	#1a	Indicate the study’s design with a commonly used term in the title or the abstract	0,1
Abstract	#1b	Provide in the abstract an informative and balanced summary	1

		of what was done and what was found	
**Introduction**			
Background / rationale	#2	Explain the scientific background and rationale for the investigation being reported	3,4
Objectives	#3	State specific objectives, including any prespecified hypotheses	5
**Methods**			
Study design	#4	Present key elements of study design early in the paper	5,6
Setting	#5	Describe the setting, locations, and relevant dates, including periods of recruitment, exposure, follow-up, and data collection	5,6
Eligibility criteria	#6a	Give the eligibility criteria, and the sources and methods of selection of participants. Describe methods of follow-up.	7
Eligibility criteria	#6b	For matched studies, give matching criteria and number of exposed and unexposed	n/a
Variables	#7	Clearly define all outcomes, exposures, predictors, potential confounders, and effect modifiers. Give diagnostic criteria, if applicable	7,8,9; table 1
Data sources / measurement	#8	For each variable of interest give sources of data and details of methods of assessment (measurement). Describe comparability of assessment methods if there is more than one group. Give information separately for for exposed and unexposed groups if applicable.	6; table 1

For peer review only

Bias	#9	Describe any efforts to address potential sources of bias	n/a
Study size	#10	Explain how the study size was arrived at	n/a
Quantitative variables	#11	Explain how quantitative variables were handled in the analyses. If applicable, describe which groupings were chosen, and why	n/a
Statistical methods	#12a	Describe all statistical methods, including those used to control for confounding	

9

Statistical methods	#12b	Describe any methods used to examine subgroups and interactions	n/a
Statistical methods	#12c	Explain how missing data were addressed	n/a
Statistical methods	#12d	If applicable, explain how loss to follow-up was addressed	n/a
Statistical methods	#12e	Describe any sensitivity analyses	

n/a

Results

Participants	#13a	Report numbers of individuals at each stage of study—eg numbers potentially eligible, examined for eligibility, confirmed eligible, included in the study, completing follow-up, and analysed. Give information separately for for exposed and	n/a

For peer review only

		unexposed groups if applicable.	
Participants	#13b	Give reasons for non-participation at each stage	n/a
Participants	#13c	Consider use of a flow diagram	
n/a			
Descriptive data	#14a	Give characteristics of study participants (eg demographic, clinical, social) and information on exposures and potential confounders. Give information separately for exposed and unexposed groups if applicable.	n/a
Descriptive data	#14b	Indicate number of participants with missing data for each variable of interest	
n/a			
Descriptive data	#14c	Summarise follow-up time (eg, average and total amount)	
n/a			
Outcome data	#15	Report numbers of outcome events or summary measures over time. Give information separately for exposed and unexposed groups if applicable.	
n/a			
Main results	#16a	Give unadjusted estimates and, if applicable, confounder-adjusted estimates and their precision (eg, 95% confidence interval). Make clear which confounders were adjusted for and why they were included	n/a
Main results	#16b	Report category boundaries when continuous variables were	n/a

For peer review only

		categorized	
Main results	#16c	If relevant, consider translating estimates of relative risk into absolute risk for a meaningful time period	

n/a

Other analyses	#17	Report other analyses done—eg analyses of subgroups and interactions, and sensitivity analyses	n/a

Discussion

Key results	#18	Summarise key results with reference to study objectives	n/a
Limitations	#19	Discuss limitations of the study, taking into account sources of potential bias or imprecision. Discuss both direction and magnitude of any potential bias.	n/a
Interpretation	#20	Give a cautious overall interpretation considering objectives, limitations, multiplicity of analyses, results from similar studies, and other relevant evidence.	n/a
Generalisability	#21	Discuss the generalisability (external validity) of the study results	n/a

Other Information

Funding	#22	Give the source of funding and the role of the funders for the present study and, if applicable, for the original study on which the present article is based	12

Notes:

- 7: 7,8,9; table 1

For peer review only

- 8: 6; table 1 The STROBE checklist is distributed under the terms of the Creative Commons Attribution License CC-BY. This checklist was completed on 18. June 2021 using https://www.goodreports.org/, a tool made by the EQUATOR Network in collaboration with Penelope.ai

For peer review only

BMJ Open

Retrospective cohort study to evaluate medication use in patients hospitalised with COVID-19 in Scotland: protocol for a national observational study

Journal:	*BMJ Open*
Manuscript ID	bmjopen-2021-054861.R1
Article Type:	Protocol
Date Submitted by the Author:	12-Oct-2021
Complete List of Authors:	Mueller, Tanja; University of Strathclyde, Strathclyde Institute of Pharmacy and Biomedical Sciences; Public Health Scotland Glasgow Office<br>Kerr, Steven; The University of Edinburgh, Usher Institute<br>McTaggart, Stuart; Public Health Scotland<br>Kurdi, Amanj; University of Strathclyde, Strathclyde Institute of Pharmacy and Biomedical Sciences; Public Health Scotland Glasgow Office<br>Vasileiou, Eleftheria; The University of Edinburgh, Usher Institute<br>Docherty, Annemarie; The University of Edinburgh, Usher Institute<br>Fraser, Kenny; Triscribe Limited<br>Shi, Ting; The University of Edinburgh, Usher Institute<br>Simpson, Colin; Victoria University of Wellington, School of Health, Wellington Faculty of Health; The University of Edinburgh, Usher Institute<br>Bennie, Marion; University of Strathclyde, Institute of Pharmacy and Biomedical Sciences; Public Health Scotland<br>Sheikh, Aziz; The University of Edinburgh, Usher Institute; HDR UK, BREATHE Hub
**Primary Subject Heading**:	Pharmacology and therapeutics
Secondary Subject Heading:	Health informatics
Keywords:	COVID-19, THERAPEUTICS, CLINICAL PHARMACOLOGY

SCHOLARONE™
Manuscripts

BMJ

I, the Submitting Author has the right to grant and does grant on behalf of all authors of the Work (as defined in the below author licence), an exclusive licence and/or a non-exclusive licence for contributions from authors who are: i) UK Crown employees; ii) where BMJ has agreed a CC-BY licence shall apply, and/or iii) in accordance with the terms applicable for US Federal Government officers or employees acting as part of their official duties; on a worldwide, perpetual, irrevocable, royalty-free basis to BMJ Publishing Group Ltd ("BMJ") its licensees and where the relevant Journal is co-owned by BMJ to the co-owners of the Journal, to publish the Work in this journal and any other BMJ products and to exploit all rights, as set out in our licence.

The Submitting Author accepts and understands that any supply made under these terms is made by BMJ to the Submitting Author unless you are acting as an employee on behalf of your employer or a postgraduate student of an affiliated institution which is paying any applicable article publishing charge ("APC") for Open Access articles. Where the Submitting Author wishes to make the Work available on an Open Access basis (and intends to pay the relevant APC), the terms of reuse of such Open Access shall be governed by a Creative Commons licence – details of these licences and which Creative Commons licence will apply to this Work are set out in our licence referred to above.

Other than as permitted in any relevant BMJ Author's Self Archiving Policies, I confirm this Work has not been accepted for publication elsewhere, is not being considered for publication elsewhere and does not duplicate material already published. I confirm all authors consent to publication of this Work and authorise the granting of this licence.

For peer review only

Retrospective cohort study to evaluate medication use in patients hospitalised with COVID-19 in Scotland: protocol for a national observational study

Authors

Tanja Mueller[1,2], Steven Kerr[3], Stuart McTaggart[4], Amanj Kurdi[1,2], Eleftheria Vasileiou[3], Annemarie Docherty[3], Kenny Fraser[5], Ting Shi[3], Colin R Simpson[3,6], Marion Bennie[1,4], Aziz Sheikh[3,7]

Affiliations

[1]Strathclyde Institute of Pharmacy and Biomedical Sciences, University of Strathclyde, Glasgow, UK

[2]Public Health Scotland, Glasgow, UK

[3]Usher Institute, University of Edinburgh, Edinburgh, UK

[4]Public Health Scotland, Edinburgh, UK

[5]Triscribe Ltd, Glasgow, UK

[6]School of Health, Wellington Faculty of Health, Victoria University of Wellington, Wellington, NZ

[7]HDR UK BREATHE Hub, Edinburgh, UK

Corresponding author

Tanja Mueller, Strathclyde Institute of Pharmacy and Biomedical Sciences, University of Strathclyde, 161 Cathedral Street, Glasgow G4 0RE, UK; email tanja.muller@strath.ac.uk

ORCID

Tanja Mueller: 0000-0002-0418-4789

Amanj Kurdi: 0000-0001-5036-1988

Eleftheria Vasileiou: 0000-0001-6850-7578

Annemarie Docherty: 0000-0001-8277-420X

Ting Shi: 0000-0002-4101-4535

Colin Simpson: 0000-0002-5194-8083

Marion Bennie: 0000-0002-4046-629x

Aziz Sheikh: 0000-0001-7022-3056

Key words: COVID-19, hospital treatment, drug utilisation, dexamethasone, remdesivir, tocilizumab

Word count: abstract 319; main text 2376 (excluding abstract, tables and references)

For peer review only

Abstract

Introduction: Coronavirus disease 2019 (COVID-19) has caused millions of hospitalisations and deaths globally. A range of vaccines have been developed and are being deployed at scale in the UK to prevent SARS-CoV-2 infection, which have reduced risk of infection and severe COVID-19 outcomes. Those with COVID-19 are now being treated with several repurposed drugs based on evidence emerging from recent clinical trials. However, there is currently limited real-world data available related to the use of these drugs in routine clinical practice. The purpose of this study is to address the prevailing knowledge gaps regarding the use of dexamethasone, remdesivir, and tocilizumab by conducting an exploratory drug utilisation study, aimed at providing in-depth descriptions of patients receiving these drugs as well as the treatment patterns observed in Scotland.

Methods and analysis: Retrospective cohort study, comprising adult patients admitted to hospital with confirmed or suspected COVID-19 across five Scottish Health Boards using data from in-hospital ePrescribing linked to the Early Estimation of Vaccine and Anti-Viral Effectiveness (EAVE II) COVID-19 surveillance platform. The primary outcome will be exposure to the medicines of interest (dexamethasone, remdesivir, tocilizumab), either alone or in combination; exposure will be described in terms of drug(s) of choice; prescribed and administered dose; treatment duration; and any changes in treatment, e.g. dose escalation and/or switching to an alternative drug. Analyses will primarily be descriptive in nature.

Ethics and dissemination: Ethical and information governance approvals have been obtained by the National Research Ethics Service Committee, South East Scotland 02, and the Public Benefit and Privacy Panel for Health and Social Care, respectively. Findings from this study will be presented at academic and clinical conferences, and to the funders and other interested parties as appropriate; study findings will also be published in peer-reviewed journals. Publications will be available on the EAVE II website (https://www.ed.ac.uk/usher/eave-ii/key-outputs/our-publications), alongside lay summaries and infographics aimed at the general public. Press releases will also be considered, if appropriate.

For peer review only - http://bmjopen.bmj.com/site/about/guidelines.xhtml

Strengths and limitations of this study

- This study will use data collected as part of routine care to address prevailing knowledge gaps with regards to the treatment of hospitalised COVID-19 patients.
- In-patient electronic prescribing data will be linked with a wide range of other datasets, enabling an in-depth description of current clinical practice in Scotland.
- Analyses will mainly be descriptive in nature; although comprising basic testing for associations between variables, causal analyses will be outwith the scope of this study due to its observational nature.

For peer review only

For peer review only - http://bmjopen.bmj.com/site/about/guidelines.xhtml

INTRODUCTION

Since first appearing in Wuhan, China, in late 2019, the new “severe acute respiratory syndrome coronavirus 2” (SARS-CoV-2) has spread globally, resulting in the World Health Organization (WHO) first declaring a Public Health Emergency of International Concern and then, in March 2020, a pandemic.[1] The disease caused by SARS-CoV-2 is now widely known as “coronavirus disease 2019”, or COVID-19.

Early symptoms of COVID-19 tend to occur between 5 – 10 days after infection, and commonly include fever, loss of smell and/or taste, and a persistent cough.[2] Symptoms may become increasingly severe over a period of approximately two weeks, and can lead to hospitalisation mainly due to breathing problems; patients with severe disease frequently require mechanical ventilation.[3] COVID-19 potentially leads to organ damage, and can result in long-term health problems (“long COVID”).[4] Disease outcomes generally appear to be linked to age and pre-existing conditions, including cardiovascular diseases and diabetes.[5]

A number of COVID-19 vaccines have been developed and are now being successfully deployed at scale in the UK.[6,7] Furthermore, while the condition itself is self-limiting in the majority of cases, a range of repurposed drugs are currently being used to alleviate symptoms and/or decrease mortality in hospitalised COVID-19 patients, mostly based on evidence emerging from clinical trials – including, e.g., antiviral drugs that have previously been tested in conditions caused by similar viruses such as SARS or MERS (Middle East respiratory syndrome) or other viral infections such as HIV or Ebola;[8,9] anti-inflammatory drugs including corticosteroids[10] and monoclonal antibodies;[11–14] and a raft of other drugs, from antibiotics[15] to interferons.[16] In addition, convalescent plasma therapy has been proposed.[17]

With interest in this area remaining high, new study results being reported on a frequent basis, and several clinical trials still ongoing, treatment recommendations are rapidly updated;[18] therefore, treatment guidelines – and, consequently, clinical practice – are likely to differ substantially, both across countries and over time. For instance, a recent multi-national cohort study has investigated the use of repurposed and adjuvant drugs in hospitalised COVID-19 patients in China, South Korea, Spain, and the United States, and found that azithromycin, the antivirals lopinavir and ritonavir, and the anti-malaria drug hydroxychloroquine were frequently used at the beginning of the pandemic; however, following reports of the non-effectiveness of these drugs in combination with safety issues related to

For peer review only - http://bmjopen.bmj.com/site/about/guidelines.xhtml

hydroxychloroquine, their use has declined, and dexamethasone and remdesivir use have instead been increasing. In addition, use patterns differed considerably between these countries.[19]

Dexamethasone,[20] remdesivir,[21] and tocilizumab[22] have been recommended for use in hospitalised patients with severe COVID-19 within the UK based on randomised controlled trial evidence, most prominently the RECOVERY trial.[23] There is, however, currently limited real-world evidence available related to the use of these drugs in routine clinical practice. For instance, it is thus far unclear which patients are being prescribed dexamethasone, remdesivir, and/or tocilizumab as part of their in-hospital treatment, and at what point; what the most common treatment patterns are; how the use of these drugs has changed since the start of the pandemic; and whether there are any geographical differences observable. Further evidence on the real-world clinical effectiveness and safety of these drugs is also required.[24]

There have been few published studies of in-hospital drug utilisation. This has been due, in part, to patient-level data being unavailable as drug prescribing and administration records are paper-based in many secondary care settings.[25] The implementation of electronic prescribing in hospitals in Scotland has simplified data sharing across health care settings. The wider roll-out of the “Hospital Electronic Prescribing and Medicines Administration” (HEPMA) system was initiated in 2014[26] in line with the Scottish eHealth strategy,[27] and HEPMA is now available to hospitals across five out of the 14 Health Boards in Scotland (regional organisations responsible for delivering health care to their respective populations).[28]

Our aim is to contribute to addressing the prevailing knowledge gaps by conducting an exploratory drug utilisation study using data from in-hospital ePrescribing linked to the Early Estimation of Vaccine and Anti-Viral Effectiveness (EAVE II) COVID-19 surveillance platform.[24] This linked data will be analysed to provide an in-depth description of the treatments hospitalised COVID-19 patients receive (whether alone or in combination), and to describe the outcomes in these patients. The drugs of interest include dexamethasone, remdesivir, and tocilizumab, based on information requests by clinicians working in this setting; these drugs are currently routinely used in patients hospitalised with COVID-19 in Scotland due to recent treatment recommendations.

For peer review only - http://bmjopen.bmj.com/site/about/guidelines.xhtml

Primary objectives

- Identify patients being treated with dexamethasone, remdesivir, and/or tocilizumab (either as monotherapy or in combination) for COVID-19 after being admitted to hospital as part of standard care;
- Describe and summarise baseline characteristics of these patients, including COVID-19 status (suspected at hospital admission based on symptoms, vs confirmed via polymerase chain reaction (PCR) test); socio-demographics (age; sex; Health Board; deprivation; hospital type; admission from care home; hospital readmission); and clinical variables potentially related to treatment choice and/or possible outcomes (including, but not restricted to, comorbidities,[29] concomitant medication, and Intensive Care Unit (ICU) admission);
- Describe treatment patterns, including drug chosen and dose administered; treatment duration; dose escalations; and changes in the drug given (e.g. switching from dexamethasone to hydrocortisone or methylprednisolone);
- Describe patterns of medicines use over time and across geographical areas, and potentially by patient characteristic as and when appropriate;
- Map out patient pathways and describe admission episodes and their outcomes (hospital admission details and duration of in-hospital stay, ICU transferal, administration of dexamethasone or any other drug of interest, discharge or death); potentially stratified by patient characteristics as and when appropriate.

Secondary objective

- Evaluate the impact of guideline changes on the patterns of use of dexamethasone, remdesivir, and tocilizumab over time.

METHODS AND ANALYSIS

Study design

Retrospective cohort study, comprising adult patients (18 years of age or older) admitted to hospital with confirmed or suspected COVID-19 across five Scottish Health Boards: NHS Ayrshire & Arran, NHS

For peer review only - http://bmjopen.bmj.com/site/about/guidelines.xhtml

Dumfries & Galloway, NHS Forth Valley, NHS Lanarkshire, and NHS Lothian. The total population size of these Health Boards was approximately 2.4m people (~45% of the Scottish population) in mid-2019.[30] However, since implementation of HEPMA in NHS Lothian happened later than in the other Health Boards, data might not be as complete, particularly for the early months of the study period.

Data sources

The data to be used for this study is part of the EAVE II platform, which has been implemented to determine COVID-19 related risk factors and the COVID-19 health care burden; and to evaluate the uptake, safety, and effectiveness of therapeutic interventions.[24] All data have been collected as part of routine care.

The EAVE II data source contains primary health care records, linked with patient-level secondary care data using the Community Health Index (CHI) number[31] – a unique patient identifier used throughout the Scottish health system – and comprises the following datasets:

- COVID-19 test results: Electronic Communication of Surveillance Scotland (ECOSS)[32]
- Vaccination status: Turas Vaccine Management Tool (TVMT),[33] GP extract
- Hospital admissions and in-patient episodes: Scottish Morbidity Record (SMR01), Rapid Preliminary Inpatient Data (RAPID), and Scottish Intensive Care Society Audit Group database (SICSAG)[34]
- In-hospital medicines use and community prescriptions: HEPMA[35] and Prescribing Information System (PIS)[34], respectively
- Mortality: National Records of Scotland (NRS)[34]

HEPMA will be used for identification of the main study outcomes, including medication use patterns; all available datasets may be used to identify other study outcomes as appropriate and feasible.

EAVE II data are held by Public Health Scotland; pseudonymised data will be accessed using the National Safe Haven, a secure, closed environment.[36]

For peer review only - http://bmjopen.bmj.com/site/about/guidelines.xhtml

Study population

The study population will comprise all adult patients admitted to hospital in the five aforementioned Health Boards since 01.03.2020 with a primary diagnosis of COVID-19, up to the latest date available. COVID-19 hospitalisations will be defined as hospitalisations within 28 days of a positive PCR test, or based on an admission with an ICD-10 code for COVID-19 (U07.1 and U07.2) as recorded in hospital episode records (SMR01 and/or RAPID); ICD-10 diagnoses will be confirmed using available PCR test results (ECOSS) where possible. Hence, the population will include both laboratory confirmed and clinical based diagnosis, respectively.

Patients will be followed up from the index date, defined as the first prescribing date for any of the medications of interest (the exposure), until discharge from hospital, death, or the end of the study period subject to data availability, whichever occurs first.

Patients receiving any of the drugs as part of a clinical trial will be excluded for analyses based on trial participation information if available (e.g. trial flag in HEPMA); or based on the dates where drugs became recommended for use in daily practice as communicated by the Scottish Government/NHS Scotland (see also Table 1 below for details).

Primary outcome

The primary outcome will be treatment with the medicines of interest, either alone or in combination, with a particular emphasis on dexamethasone as dexamethasone is the most widely used of these drugs, and the availability of both prescribing and administration data is expected to be high. All patients receiving dexamethasone will be included in the first instance; however, analyses will mainly focus on those receiving the recommended dosing regimen for patients with COVID-10 (6mg po or 1.8ml iv, once daily for 10 days)[20] since other dosing regimens are more likely being prescribed for indications other than COVID-19. Alternative recommended corticosteroids such as prednisolone and hydrocortisone will also be considered (recommended doses: 40mg po once daily for 10 days; 50mg iv every eight hours for 10 days, respectively). Remdesivir and tocilizumab will be included for analyses where sufficient data are available; since these two drugs are given intravenously, the data available might be limited (i.e. exclude the exact dose administered).

Exposure will be described in terms of drug(s) of choice; prescribed and administered dose; treatment duration; and any changes in treatment, e.g. dose escalation and/or switching to an alternative drug

For peer review only - http://bmjopen.bmj.com/site/about/guidelines.xhtml

(including time to dose escalation/switching and reasons for these, if available). The first drug prescribed on HEPMA following admission to hospital on or after the date of (possible) COVID-19 diagnosis will be defined as the index drug (i.e. dexamethasone, remdesivir, or tocilizumab); the date of the first recorded prescription will be used as the index date (for the purpose of setting the baseline). Duration of treatment will be calculated using the dates of first/last recorded administration of the drug in question.

Other outcomes

Additional outcomes relating to the primary study objective include hospital specialty at admission, in-hospital transfer (e.g. admission to ICU), length of stay, and outcome of hospital episode (discharge or death).

Secondary outcomes include in-hospital mortality, i.e. death on the same day as discharge; and out-of-hospital mortality following discharge, if feasible.

Covariates

Patient characteristics of interest that might potentially influence choice of drug, duration of treatment, and (possibly) treatment outcomes will be identified and summarised at baseline, and comprise socio-demographic factors (age, sex, Health Board of residence, level of deprivation); disease-related aspects; comorbidities; and concomitant medication.

The level of deprivation will be characterised using the Scottish Index of Multiple Deprivation (SIMD), an area index combining information with regards to health, access to services, education, employment, income, housing, and crime.[37] Disease-related aspects refer to information potentially linked to disease severity, e.g. level of hospital care/additional treatments received (ICU admission, mechanical ventilation) if and where available; while O_2 saturation levels would be highly relevant, particularly with regards to treatment outcomes, this information is not present in the available dataset. Comorbidities of interest will comprise mainly those conditions used to identify patients at high risk of adverse outcomes (i.e. shielding list),[29] e.g. respiratory disease (asthma, chronic obstructive pulmonary disease (COPD)), cardiovascular diseases, diabetes (type 1 and type 2), chronic kidney disease, and cancer; other comorbidities might also be included. Concomitant medication at baseline will focus on drugs potentially impacting the immune system and/or affecting the risk of infections (immuno-suppressants, steroids, antimicrobial drugs), and those with an (hypothesised) effect on disease severity or outcome – either directly or as a proxy for underlying conditions potentially not captured otherwise within the dataset

For peer review only - http://bmjopen.bmj.com/site/about/guidelines.xhtml

(e.g. long-acting muscarinic antagonists (LAMA)/long acting beta-agonists (LABA), insulin and anti-diabetic drugs, anticoagulants, antiplatelet drugs, angiotensin-converting enzyme (ACE) inhibitors, angiotensin receptor blockers (ARBs)). If possible, COVID-19 vaccination status of patients will also be assessed. Furthermore, the presence of polypharmacy – defined as the simultaneous use of five or more different medications prior to being admitted to hospital – will be identified.

Baseline characteristics will be defined using all available data, with restrictions on included time periods (mainly with regards to concomitant medication) based on the specifications used in previous studies.[5,6,24]

Exposure, outcome, and relevant covariates – alongside the data source, their coding, and a brief description – are presented in table 1.

Table 1: Description of variables (cohort identification, outcomes, covariates)

**Variable**	**Data source**	**Description**	**Value**
Cohort: COVID-19 status			
Cause of admission	SMR01/ RAPID	Suspected or confirmed	ICD10 codes: U07.1, U07.2
PCR test result	ECOSS	COVID-19 test result (within 28 days prior to admission)	Categorical: Positive, negative, unavailable
Primary outcome: medication use			
Drug name	HEPMA	Drugs of interest: Dexamethasone, remdesivir, tocilizumab [1]	Character (name, according to dm+d)
Drug dose	HEPMA	Prescribed and administered	Numeric (mg, ml)
Prescribed date	HEPMA	First prescribed date: index date	Date (yyyy-mm-dd)
Administered date	HEPMA	Dates of drug administration	Date (yyyy-mm-dd)
Duration of treatment *	HEPMA	(first – last administered date) [2]	Numeric (days)
Treatment changes *	HEPMA	Changes in dosing and/or drug	Categorical: yes, no
Secondary outcomes			
Hospital specialty	SMR01	At admission	Character (name)
Specialty changes *	SMR01	Internal transferals during stay	Categorical: yes, no

For peer review only - http://bmjopen.bmj.com/site/about/guidelines.xhtml

ICU/HDU	SICSAG	Admission to intensive care	Categorical: yes, no
Discharge: alive	SMR01	Outcome of hospital episode	Categorical: home, w/family, care facility, other hospital
Discharge: dead	SMR01, NRS	Outcome of hospital admission (In-hospital mortality)	Categorical: yes, no<br>Cause: ICD-10 codes
Death	NRS	Overall mortality (after discharge)	Categorical: yes, no<br>Cause: ICD-10 codes
Length of stay [3]	SMR01	Duration of in-hospital stay	Numeric (days)
Covariates: socio-demographic			
Age *	GP extract	Patient age at index date	Numeric (years)
Sex	GP extract	Biological sex at birth	Categorical: male, female
Health Board	GP extract	Patient place of residence at admission	Categorical: A&A, D&G, Forth Valley, Lanarkshire, Lothian;
Data zone	GP extract	Patient place of residence	Categorical
SIMD	GP extract	Level of deprivation, based on data zone of residence	Categorical: 1 (most) to 5 (least deprived)
Covariates: disease (severity) related			
COVID-19 vaccination status	GP extract/ TVMT	Status at hospital admission	Categorical: unvaccinated, vaccinated once, twice
Level of care	SICSAG/ SMR01	Admission to ICU; level of care received while at ICU	Categorical: yes, no<br>Categorical: ACP levels 0-3
Supporting medication	HEPMA	Therapeutics prescribed and administered during in-hospital stay	Character (name, according to dm+d)
Covariates: comorbidities and concomitant medication			
Other causes of admission	SMR01	Conditions underlying or attributing to hospital admission	ICD-10 codes
Comorbidities	GP extract/ SMR01	Pre-existing conditions	READ codes, ICD-10 codes
Charlson score * [38]	SMR01	Estimated based on secondary care data (historic hospital episodes)	Numeric
Concomitant medication	PIS	Potential proxy for comorbidities; specific drugs of interest	Character (name, according to the BNF)
Polypharmacy *	PIS	Based on number of different drugs prescribed simultaneously	Categorical: yes, no

For peer review only

For peer review only - http://bmjopen.bmj.com/site/about/guidelines.xhtml

* denotes derived variables

[1] Cut-off dates to exclude patients who have been treated as part of a clinical trial, if no trial flag participation available in the dataset: remdesivir 29.05.2020; dexamethasone16.06.2020; tocilizumab 08.01.2021
[2] Adding discharge/outpatient prescribing if patient discharged prior to end of treatment regimen (if available)
[3] Can be derived if variable not readily available in dataset (date of discharge – first date of admission)

ACP - Augmented Care Period; BNF - British National Formulary; dm+d - Dictionary of Medicines and Devices; ECOSS - Electronic Communication of Surveillance in Scotland; HDU - High Dependency Unit; HEPMA - Hospital Electronic Prescribing and Medicines Administration; ICD-10 - International Classification of Diseases, 10th Edition; ICU - Intensive Care Unit; NRS - National Records of Scotland; PCR - polymerase chain reaction; PIS - Prescribing Information System; RAPID - rapid preliminary in-patient data; SICSAG - Scottish Intensive Care Society Audit Group; SIMD - Scottish Index of Multiple Deprivation; SMR01 - Scottish Morbidity Records, inpatient dataset; TVMT - Tuas Vaccine Management Tool

Statistical analysis

All analyses relating to the primary objectives of this study will be descriptive in nature, and may include counts/frequencies for categorical variables, and mean/SD or median/IQR for continuous variables, as appropriate. In addition, patient pathways will be visualised using Sankey plots or similar techniques.

The impact of changes in treatment guidelines on the use of dexamethasone will be evaluated using interrupted time series analysis; logistic regression or time-to-event analysis (e.g. Kaplan-Meier plots) will be used to assess discharge patterns or patient mortality, if feasible.

All analyses will be conducted using R/RStudio, version 3.6.1.[39,40]

Patient and public involvement

The EAVE II Public Advisory group are a diverse group of PPI contributors who meet monthly to incorporate the views of patients and the public into research using the EAVE II dataset. This includes shaping of research via the EAVE II Steering Group, which is attended by our two lay leads. The lay summary for this research will be co-written with our PPI contributors and shared via the outputs section of the EAVE II website,[36] hosted by the University of Edinburgh.

ETHICS AND DISSEMINATION

Ethical and information governance approvals have been obtained by the National Research Ethics Service Committee (REC), South East Scotland 02 (REC number: 12/SS/0201), and the Public Benefit and Privacy Panel for Health and Social Care (reference number: 1920-0279) respectively. Findings from this study will be presented at academic and clinical conferences, and to the funders and other interested parties as appropriate. Study findings will also be published in peer-reviewed journals; reporting of

For peer review only - http://bmjopen.bmj.com/site/about/guidelines.xhtml

findings will follow the STROBE (Strengthening the Reporting of Observational Studies in Epidemiology)[41] and RECORD (Reporting of Studies conducted using Observational Routinely-collected Data) guidelines.

Acknowledgements: We thank Dave Kelly from Albasoft Ltd for his support with making primary care data available; and Wendy Inglis-Humphrey and Vicky Hammersley for their support with project management and administration. This work is only possible because of the wealth of information collected by the NHS as part of routine clinical practice.

Authors' contributions: TM, SK, SM, AK and MB conceptualised the study. EV, AD, KF, TS and CS provided additional methodological and/or clinical advice. AS is the principal investigator of the EAVE II project and provides strategic advice. TM drafted the protocol. All authors read, critically revised, and approved the final draft.

Funding: This analysis is part of the Early Assessment of COVID-19 epidemiology and Vaccine/anti-viral Effectiveness (EAVE II) study. EAVE II is funded by the Medical Research Council [MC_PC_19075] with the support of BREATHE – The Health Data Research Hub for Respiratory Health [MC_PC_19004], which is funded through the UK Research and Innovation Industrial Strategy Challenge Fund and delivered through Health Data Research UK. Additional support has been provided through the Scottish Government DG Health and Social Care.

Competing interests: AS reports grants from NIHR, grants from MRC, grants from HDR UK, during the conduct of the study. CS reports funding from NIHR (UK), MRC (UK), CSO (UK), Health Research Council (NZ), and Ministry for Business, Innovation and Employment (NZ) during the conduct of this study. KF is Director of Triscribe Ltd, a company providing data quality services and software support. All other authors report no conflicts of interest.

Data availability statement: NHS data is confidential, and is only available upon request subject to approval by a Caldicott Guardian/the Public Benefit and Privacy Panel for Health and Social Care.

Patient consent for publication: Not required.

For peer review only - http://bmjopen.bmj.com/site/about/guidelines.xhtml

REFERENCES

1 WHO. Coronavirus disease (COVID-19) – World Health Organization. 2021.https://www.who.int/emergencies/diseases/novel-coronavirus-2019 (accessed 8 Jun 2021).

2 NHS 24. Coronavirus (COVID-19) in Scotland | NHS inform. 2021.https://www.nhsinform.scot/illnesses-and-conditions/infections-and-poisoning/coronavirus-covid-19 (accessed 8 Jun 2021).

3 Kevadiya BD, Machhi J, Herskovitz J, *et al.* Pharmacotherapeutics of SARS-CoV-2 Infections. *J Neuroimmune Pharmacol* 2021;:1–26. doi:10.1007/s11481-020-09968-x

4 Iacobucci G. Long covid: Damage to multiple organs presents in young, low risk patients. *BMJ* 2020;**371**:m4470. doi:10.1136/bmj.m4470

5 Clift AK, Coupland CAC, Keogh RH, *et al.* Living risk prediction algorithm (QCOVID) for risk of hospital admission and mortality from coronavirus 19 in adults: national derivation and validation cohort study. *BMJ* 2020;**371**:m3731. doi:10.1136/bmj.m3731

6 Vasileiou E, Simpson CR, Shi T, *et al.* Interim findings from first-dose mass COVID-19 vaccination roll-out and COVID-19 hospital admissions in Scotland: a national prospective cohort study. *The Lancet* 2021;**397**:1646–57. doi:10.1016/S0140-6736(21)00677-2

7 Vasileiou E, Simpson CR, Robertson C, *et al.* Effectiveness of First Dose of COVID-19 Vaccines Against Hospital Admissions in Scotland: National Prospective Cohort Study of 5.4 Million People. Rochester, NY: : Social Science Research Network 2021. doi:10.2139/ssrn.3789264

8 Beigel JH, Tomashek KM, Dodd LE, *et al.* Remdesivir for the Treatment of Covid-19 — Final Report. *New England Journal of Medicine* Published Online First: 22 May 2020. doi:10.1056/NEJMoa2007764

9 Horby PW, Mafham M, Bell JL, *et al.* Lopinavir–ritonavir in patients admitted to hospital with COVID-19 (RECOVERY): a randomised, controlled, open-label, platform trial. *The Lancet* 2020;**396**:1345–52. doi:10.1016/S0140-6736(20)32013-4

10 RECOVERY Collaborative Group, Horby P, Lim WS, *et al.* Dexamethasone in Hospitalized Patients with Covid-19. *N Engl J Med* 2021;**384**:693–704. doi:10.1056/NEJMoa2021436

11 REMAP-CAP Investigators. Interleukin-6 Receptor Antagonists in Critically Ill Patients with Covid-19. *New England Journal of Medicine* 2021;**384**:1491–502. doi:10.1056/NEJMoa2100433

12 RECOVERY Collaborative Group, Horby PW, Pessoa-Amorim G, *et al.* Tocilizumab in patients admitted to hospital with COVID-19 (RECOVERY): preliminary results of a randomised, controlled, open-label, platform trial. *medRxiv* 2021;:2021.02.11.21249258. doi:10.1101/2021.02.11.21249258

13 Weinreich DM, Sivapalasingam S, Norton T, *et al.* REGN-COV2, a Neutralizing Antibody Cocktail, in Outpatients with Covid-19. *New England Journal of Medicine* 2021;**384**:238–51. doi:10.1056/NEJMoa2035002

14 RECOVERY Collaborative Group, Horby PW, Mafham M, *et al.* Casirivimab and imdevimab in patients admitted to hospital with COVID-19 (RECOVERY): a randomised, controlled, open-label, platform trial. *medRxiv* 2021;:2021.06.15.21258542. doi:10.1101/2021.06.15.21258542

For peer review only - http://bmjopen.bmj.com/site/about/guidelines.xhtml

15 Abaleke E, Abbas M, Abbasi S, *et al.* Azithromycin in patients admitted to hospital with COVID-19 (RECOVERY): a randomised, controlled, open-label, platform trial. *The Lancet* 2021;**397**:605–12. doi:10.1016/S0140-6736(21)00149-5

16 Monk PD, Marsden RJ, Tear VJ, *et al.* Safety and efficacy of inhaled nebulised interferon beta-1a (SNG001) for treatment of SARS-CoV-2 infection: a randomised, double-blind, placebo-controlled, phase 2 trial. *The Lancet Respiratory Medicine* 2021;**9**:196–206. doi:10.1016/S2213-2600(20)30511-7

17 Simonovich VA, Burgos Pratx LD, Scibona P, *et al.* A Randomized Trial of Convalescent Plasma in Covid-19 Severe Pneumonia. *New England Journal of Medicine* 2021;**384**:619–29. doi:10.1056/NEJMoa2031304

18 National Institute for Health and Care Excellence. Recommendations | COVID-19 rapid guideline: managing COVID-19 | Guidance | NICE. https://www.nice.org.uk/guidance/ng191/chapter/Recommendations (accessed 21 Jun 2021).

19 Prats-Uribe A, Sena AG, Lai LYH, *et al.* Use of repurposed and adjuvant drugs in hospital patients with covid-19: multinational network cohort study. *BMJ* 2021;**373**:n1038. doi:10.1136/bmj.n1038

20 Scottish Government. COVID-19 therapeutic alert: Dexamethasone in the treatment of COVID-19: implementation and management of supply for treatment in hospitals. https://www.sehd.scot.nhs.uk/publications/DC20200616COVID-19Dexamethasone.pdf (accessed 6 Aug 2021).

21 NHS England. Coronavirus » Interim Clinical Commissioning Policy: Remdesivir for patients hospitalised with COVID-19 (adults and children 12 years and older). https://www.england.nhs.uk/coronavirus/publication/interim-clinical-commissioning-policy-remdesivir-for-patients-hospitalised-with-covid-19-adults-and-children-12-years-and-older/ (accessed 23 Mar 2021).

22 NHS England. Coronavirus » Interim Clinical Commissioning Policy: Tocilizumab for hospitalised patients with COVID-19 pneumonia (adults). https://www.england.nhs.uk/coronavirus/publication/interim-clinical-commissioning-policy-tocilizumab-for-hospitalised-patients-patients-with-covid-19-pneumonia-adults/ (accessed 23 Mar 2021).

23 Nuffield Department of Population Health. RECOVERY - Randomised Evaluation of COVID-19 Therapy. 2021.https://www.recoverytrial.net/ (accessed 8 Jun 2021).

24 Simpson CR, Robertson C, Vasileiou E, *et al.* Early Pandemic Evaluation and Enhanced Surveillance of COVID-19 (EAVE II): protocol for an observational study using linked Scottish national data. *BMJ Open* 2020;**10**:e039097. doi:10.1136/bmjopen-2020-039097

25 Warren LR, Clarke J, Arora S, *et al.* Improving data sharing between acute hospitals in England: an overview of health record system distribution and retrospective observational analysis of inter-hospital transitions of care. *BMJ Open* 2019;**9**:e031637. doi:10.1136/bmjopen-2019-031637

26 NHS Scotland. HEPMA | eHealth. https://www.ehealth.scot/case-studies/hepma/ (accessed 8 Jun 2021).

27 Scottish Government. eHealth Strategy 2014-2017. Edinburgh, UK: : Scottish Government 2015. https://www.gov.scot/publications/ehealth-strategy-2014-2017/ (accessed 15 Oct 2020).

28 NHS Scotland. Organisations – Scotland's Health on the Web. 2020.https://www.scot.nhs.uk/organisations/ (accessed 8 Jun 2021).

For peer review only

For peer review only - http://bmjopen.bmj.com/site/about/guidelines.xhtml

29 NHS Digital. Rule logic. NHS Digital. https://digital.nhs.uk/coronavirus/shielded-patient-list/methodology/rule-logic (accessed 7 Jun 2021).

30 Public Health Scotland. Population Estimates - Scottish Health and Social Care Open Data. 2021.https://www.opendata.nhs.scot/dataset/population-estimates (accessed 14 Jun 2021).

31 Information Services Division. Data Dictionary A-Z: CHI number. ISD Scotland Data Dictionary. https://www.ndc.scot.nhs.uk/Dictionary-A-Z/Definitions/index.asp?ID=128&Title=CHI%20Number (accessed 15 Oct 2020).

32 Health Protection Scotland. Data and surveillance. https://www.hps.scot.nhs.uk/data/ (accessed 8 Jun 2021).

33 NHS Education for Scotland. Turas Vaccination Management tool. 2021.https://learn.nes.nhs.scot/42708/turas-vaccination-management-tool (accessed 8 Jun 2021).

34 Information Services Division. National Data Catalogue: National Datasets. ISD Scotland National Data Catalogue. https://www.ndc.scot.nhs.uk/National-Datasets/index.asp (accessed 15 Oct 2020).

35 NHS Digital. Hospital Electronic Prescribing and Medicines Administration (HEPMA) Data - Scotland. NHS Digital. https://digital.nhs.uk/about-nhs-digital/corporate-information-and-documents/directions-and-data-provision-notices/data-provision-notices-dpns/electronic-prescribing-and-medicines-administration-data-scotland (accessed 8 Jun 2021).

36 University of Edinburgh. EAVE II. The University of Edinburgh. https://www.ed.ac.uk/usher/eave-ii (accessed 8 Jun 2021).

37 Scottish Government. The Scottish Index of Multiple Deprivation. Statistics. 2020.https://www2.gov.scot/SIMD (accessed 15 Oct 2020).

38 Quan H, Sundararajan V, Halfon P, *et al.* Coding algorithms for defining comorbidities in ICD-9-CM and ICD-10 administrative data. *Med Care* 2005;**43**:1130–9. doi:10.1097/01.mlr.0000182534.19832.83

39 R Core Team. *R: A language and environment for statistical computing*. Vienna, Austria: : R Foundation for Statistical Computing 2020. https://www.R-project.org/

40 RStudio Team. *RStudio: Integrated Development for R*. Boston, MA: : RStudio, PBC 2020. http://www.rstudio.com/

41 Elm E von, Altman DG, Egger M, *et al.* Strengthening the reporting of observational studies in epidemiology (STROBE) statement: guidelines for reporting observational studies. *BMJ* 2007;**335**:806–8. doi:10.1136/bmj.39335.541782.AD

For peer review only - http://bmjopen.bmj.com/site/about/guidelines.xhtml

Reporting checklist for cohort study.

Based on the STROBE cohort guidelines.

Instructions to authors

Complete this checklist by entering the page numbers from your manuscript where readers will find each of the items listed below.

Your article may not currently address all the items on the checklist. Please modify your text to include the missing information. If you are certain that an item does not apply, please write "n/a" and provide a short explanation.

Upload your completed checklist as an extra file when you submit to a journal.

In your methods section, say that you used the STROBE cohortreporting guidelines, and cite them as:

von Elm E, Altman DG, Egger M, Pocock SJ, Gotzsche PC, Vandenbroucke JP. The Strengthening the Reporting of Observational Studies in Epidemiology (STROBE) Statement: guidelines for reporting observational studies.

For peer review only

		Reporting Item	Page Number
**Title and abstract**			
Title	#1a	Indicate the study’s design with a commonly used term in the title or the abstract	0,1
Abstract	#1b	Provide in the abstract an informative and balanced summary	1

		of what was done and what was found	
**Introduction**			
Background / rationale	#2	Explain the scientific background and rationale for the investigation being reported	3,4
Objectives	#3	State specific objectives, including any prespecified hypotheses	5
**Methods**			
Study design	#4	Present key elements of study design early in the paper	5,6
Setting	#5	Describe the setting, locations, and relevant dates, including periods of recruitment, exposure, follow-up, and data collection	5,6
Eligibility criteria	#6a	Give the eligibility criteria, and the sources and methods of selection of participants. Describe methods of follow-up.	7
Eligibility criteria	#6b	For matched studies, give matching criteria and number of exposed and unexposed	n/a
Variables	#7	Clearly define all outcomes, exposures, predictors, potential confounders, and effect modifiers. Give diagnostic criteria, if applicable	7,8,9; table 1
Data sources / measurement	#8	For each variable of interest give sources of data and details of methods of assessment (measurement). Describe comparability of assessment methods if there is more than one group. Give information separately for for exposed and unexposed groups if applicable.	6; table 1

For peer review only

Bias	#9	Describe any efforts to address potential sources of bias	n/a
Study size	#10	Explain how the study size was arrived at	n/a
Quantitative variables	#11	Explain how quantitative variables were handled in the analyses. If applicable, describe which groupings were chosen, and why	n/a
Statistical methods	#12a	Describe all statistical methods, including those used to control for confounding	
9			
Statistical methods	#12b	Describe any methods used to examine subgroups and interactions	n/a
Statistical methods	#12c	Explain how missing data were addressed	n/a
Statistical methods	#12d	If applicable, explain how loss to follow-up was addressed	n/a
Statistical methods	#12e	Describe any sensitivity analyses	
n/a			

Results

Participants	#13a	Report numbers of individuals at each stage of study—eg numbers potentially eligible, examined for eligibility, confirmed eligible, included in the study, completing follow-up, and analysed. Give information separately for for exposed and	n/a

For peer review only

		unexposed groups if applicable.	
Participants	#13b	Give reasons for non-participation at each stage	n/a
Participants	#13c	Consider use of a flow diagram	
n/a			
Descriptive data	#14a	Give characteristics of study participants (eg demographic, clinical, social) and information on exposures and potential confounders. Give information separately for exposed and unexposed groups if applicable.	n/a
Descriptive data	#14b	Indicate number of participants with missing data for each variable of interest	
n/a			
Descriptive data	#14c	Summarise follow-up time (eg, average and total amount)	
n/a			
Outcome data	#15	Report numbers of outcome events or summary measures over time. Give information separately for exposed and unexposed groups if applicable.	
n/a			
Main results	#16a	Give unadjusted estimates and, if applicable, confounder-adjusted estimates and their precision (eg, 95% confidence interval). Make clear which confounders were adjusted for and why they were included	n/a
Main results	#16b	Report category boundaries when continuous variables were	n/a

For peer review only

		categorized	
Main results	#16c	If relevant, consider translating estimates of relative risk into absolute risk for a meaningful time period	
n/a			
Other analyses	#17	Report other analyses done—eg analyses of subgroups and interactions, and sensitivity analyses	n/a
**Discussion**			
Key results	#18	Summarise key results with reference to study objectives	n/a
Limitations	#19	Discuss limitations of the study, taking into account sources of potential bias or imprecision. Discuss both direction and magnitude of any potential bias.	n/a
Interpretation	#20	Give a cautious overall interpretation considering objectives, limitations, multiplicity of analyses, results from similar studies, and other relevant evidence.	n/a
Generalisability	#21	Discuss the generalisability (external validity) of the study results	n/a
**Other Information**			
Funding	#22	Give the source of funding and the role of the funders for the present study and, if applicable, for the original study on which the present article is based	12

Notes:

- 7: 7,8,9; table 1

For peer review only

- 8: 6; table 1 The STROBE checklist is distributed under the terms of the Creative Commons Attribution License CC-BY. This checklist was completed on 18. June 2021 using https://www.goodreports.org/, a tool made by the EQUATOR Network in collaboration with Penelope.ai

For peer review only
